# Nodal Dynamics, Not Degree Distributions, Determine the Structural Controllability of Complex Networks

**DOI:** 10.1371/journal.pone.0038398

**Published:** 2012-06-22

**Authors:** Noah J. Cowan, Erick J. Chastain, Daril A. Vilhena, James S. Freudenberg, Carl T. Bergstrom

**Affiliations:** 1 Department of Mechanical Engineering, Johns Hopkins University, Baltimore, Maryland, United States of America; 2 Department of Computer Science, Rutgers University, New Brunswick, New Jersey, United States of America; 3 Department of Biology, University of Washington, Seattle, Washington, United States of America; 4 Department of Electrical Engineering and Computer Science, University of Michigan, Ann Arbor, Michigan, United States of America; 5 Santa Fe Institute, Santa Fe, New Mexico, United States of America; Queen’s University Belfast, United Kingdom

## Abstract

Structural controllability has been proposed as an analytical framework for making predictions regarding the control of complex networks across myriad disciplines in the physical and life sciences (Liu et al., Nature:473(7346):167–173, 2011). Although the integration of control theory and network analysis is important, we argue that the application of the structural controllability framework to most if not all real-world networks leads to the conclusion that a single control input, applied to the power dominating set, is all that is needed for structural controllability. This result is consistent with the well-known fact that controllability and its dual observability are generic properties of systems. We argue that more important than issues of structural controllability are the questions of whether a system is almost uncontrollable, whether it is almost unobservable, and whether it possesses almost pole-zero cancellations.

## Introduction

How can we control complex networks of dynamical systems [1]–[9]? Is it sufficient to control a few nodes, or are inputs needed at a large fraction of the nodes in the network? Which nodes need to be controlled? A recent paper [10] suggests that we can address these problems using the concept of structural controllability [11], and in doing so we may be able to forge new connections between control theory and complex networks. The two main results from this analysis are (1) that the number of driver nodes, 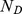, necessary to control a network is determined by the network’s degree distribution and (2) that 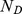 tends to comprise a substantial fraction of the nodes in inhomogeneous networks such as the real-world examples considered therein.

However, both conclusions hinge on a critical assumption of the model in [10]: the results (implicitly) require that the “default” structures of the dynamical systems at the nodes of the network have infinite time constants. This modeling assumption implies that, unless otherwise specified by a self-link in the network, a node’s state never changes absent influence from inbound connections. However, the real networks considered in [10]–including food webs, power grids, electronic circuits, regulatory networks, and neuronal networks–typically manifest more general dynamics at each node, i.e. they typically have finite time constants [12]–[14].

With this assumption, the minimum number of independent control inputs required to ensure a technical property known as *structural controllability* [11] can be calculated for the network, as described in [10]. The main problem with the argument set forth in [10] is not a technical one: indeed the assumptions therein are clear and the mathematical results are correct. Then, why are the results tenuous? Critically, structural controllability [11] is premised on the idea that if the *nonzero* parameters in the mathematical model can be selected so that the system is controllable (an elementary concept in control theory; see for example [15]), then the system will be controllable for all parameters except a set of zero measure. That is, if the system is controllable for one set of (initially nonzero) parameters, then controllability is guaranteed generically for that system. The results presented in [10] require that a critical assumption be made *before* applying the structural controllability approach. Specifically, it is assumed that each node has an infinite time constant. As we shall see in the next section, the assumption of an infinite time constant implies that a certain parameter in the mathematical model of the system is equal to zero, and therefore that term is off-limits as far as structural controllability is concerned. As one can imagine, any approach to system analysis that only allows the modification of nonzero terms, makes the results potentially quite sensitive to which terms are set to zero in the first place. Indeed, if the infinite-time-constant assumption is relaxed, and generic linear dynamics are ascribed to each node, one obtains a categorically different result. Indeed, we show in this paper that all networks with finite-dimensional linear dynamics (save a special set of parameters of zero measure) are controllable with a single input. While mathematically true, such a conclusion is neither reasonable nor practical for real-world networks, and thus calls into question the general approach of applying structural controllability in this way.

Assuming arbitrary (up to a set of measure zero) linear dynamics, we show here that (1) a single time-dependent input is all that is needed for structural controllability, and (2) that this input should be applied to the power dominating set (PDS) [16] of the network. Thus for many if not all naturally occurring network systems, structural controllability does not depend on degree distribution and can always be conferred with a single control input.

## Results

### Modeling Networks for Control

Large interconnected systems are commonly represented as complex networks [17], [18]. For many biological and physical networks, each node in the network corresponds to a dynamical system. Often, the dynamics of these nodes can be modeled by a system of ordinary differential equations [19], [20]:
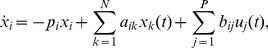
(1)where 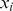 is a state at node 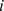, 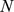 is the number of nodes, 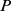 is the number of inputs, and the 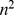 elements 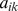 populate the adjacency matrix. Here, the term 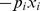 represents the intrinsic dynamics at the node, absent external influences. The external inputs, 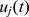, enter the system through the coupling matrix 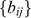. For analyzing controllability, it is reasonable as a first step to consider purely linear dynamics as shown in Eq. (1)—an approach clearly articulated and well motivated by [10].

Note that Eq. (1) includes two terms in dynamics for 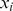, one related to the linearization of the intrinsic nodal dynamics, namely 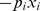, and one related to a potential self link in the model, namely 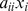, related to the network topology. Although both terms are identical mathematically, they arise from categorically different sources, and thus are not interchangeable.

The term 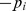 is the *pole* of the linear dynamical system at each node, and 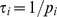 is the associated time constant. Rewriting in terms of transfer functions, we have.
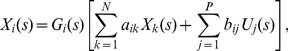
(2)where 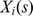 and 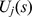 are the Laplace transforms of state 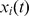 and input 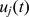 respectively, and



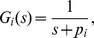



is the transfer function of node 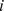. This formulation is useful because it suggests inclusion of more general linear dynamics: the transfer function, 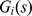, can be replaced by any transfer function, of arbitrary order.

The dynamics proposed in [10] (see the supplemental material therein) are identical to (2), except that 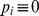 for all 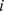, namely 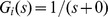
–a pure integrator. Written this way the simplifying assumption of the model in [10] becomes clear: all subsystems by default have an infinite time constants (that is, the term 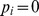) unless such dynamics are explicitly included in the network data set through nonzero diagonal terms, 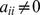, in the adjacency matrix.

However, infinite time constants at each node do not generally reflect the dynamics of the physical and biological systems in Table 1 of [10]. Reproduction and mortality schedules imply species-specific time constants in trophic networks. Molecular products spontaneously degrade at different rates in protein interaction networks and gene regulatory networks. Absent synaptic input, neuronal activity returns to baseline at cell-specific rates. Indeed, most if not all systems in physics, biology, chemistry, ecology, and engineering will have a linearization with a finite time constant. Thus while the model in [10] does not proscribe self-links, this approach does place the onus on the modeler to ensure that any network representation includes such self-links where appropriate to compensate for the omission of the *intrinsic* nodal dynamics that arise due to physical, biological, or other processes that, generally speaking, have nothing to do with network topology.

To see the consequences of including generic nodal dynamics on a network’s structural controllability, we first rewrite the network dynamics in (2) in state space form:
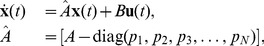
(3)where 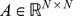 is the adjacency matrix, and 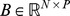 is the input matrix. The vector 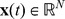 is the vector of node states, and 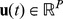 is the input vector.

The system in Eq. (3) is controllable if and only if the matrix.
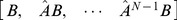
(4)


is full rank, a standard result in control theory [15]. The system is said to be *structurally controllable* if the nonzero weights in 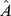 and 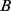 can be adjusted such that the matrix in Eq. (4) is full rank [11].

In [10], the minimum number of driver nodes, 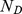, is defined as the minimum number of inputs—i.e., independent, user defined, time-varying functions—such that when injected into the network guarantee structural controllability. This formulation explicitly allows each independent input to be connected to multiple (and possibly all) nodes in the network [10], [21].

The paper [10] solves this minimum input problem using an application of graph-theoretic concepts; their basic approach is to identify the number of “unmatched nodes” after finding a so-called maximum matching of the graph. Details are provided in the supplemental material of [10]; note also the prior analysis wherein the maximum matching theorem seems first to have been proved [22]. We observe that one can recast the poles at 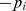 as (nonzero) self-links. But the set of all self-links 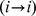 is itself a maximum matching; all nodes in the network are then matched nodes. This implies that the network can be controlled with a single input, i.e. 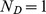, which follows directly from the maximum matching proof in [10].

### Structural Controllability of Networks with General Linear Dynamics

The following proposition provides a simple non-graph-theoretic proof that a “control hub” – a single driver node attached to *all nodes* – guarantees structural controllability with a single input.

**Proposition 1** For any directed network with nodal dynamics in Eq. 2 (or equivalently Eq. 3), with 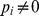 and/or 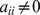, 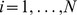, then 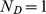.

*Proof.* Select 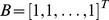 (that is, connect a single input to all nodes). Lin’s structural controllability theorem [11] states that if the system is controllable for one choice of the nonzero system parameters, then it will be controllable for all parameters except a set of measure zero. So, we explicitly construct a parameter set that makes the system controllable. Keep 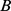 as all ones, and choose 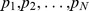 to be nonzero and distinct. Zero out all the network edges (i.e. nullify the adjacency matrix, 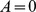). The system matrix 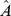 is now a diagonal matrix with distinct eigenvalues. Controllability of 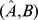 follows by inspection. Thus, the system is structurally controllable and 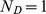.

By contrast the paper [10] reported that for real-world networks, the minimum number of driver nodes 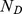 is strongly influenced by the sparseness and homogeneity of the network, as measured by the *degree distribution*, 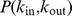 (see [10] for more details). Why did [10] arrive at such different conclusions? Critically, the application of structural controllability does not consider variations in system parameters that are *a priori* zero [11]. So, for example, if a link 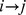 is absent, then 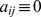. The original paper [10] allows for self-links but by default does not include them. Further, the framework set forth in [10] assumes 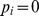 (infinite time constant), and the network datasets in Table 1 of [10] do not include self-links to correct for this. Therefore, upon inclusion of first-order self dynamics, essentially all real networks are structurally controllable with 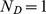, irrespective of network topology.

In the case that the network topology does not explicitly contain self links, the consequence of ascribing pure integrator dynamics (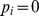) to each node is categorical: the system is necessarily unstable. This is because the sum of the eigenvalues is given by the trace of the system matrix, which, in this case, would be 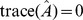, since there are zeros on the entire diagonal. This would imply that it is impossible to have any stable eigenvalues (negative real parts) without also having unstable ones (positive real parts), so that their sum is zero. Therefore, such a network of integrators must be purely oscillatory or unstable, and cannot be asymptotically stable. Therefore, assuming pure integrators at each node, and no explicit self-links in the adjacency matrix, precludes passive stability which many natural systems enjoy.

Have we taken the point about generic nodal dynamics too far? It may be desirable to model and control a network on a timescale that is faster than the dynamics of the intrinsic nodal dynamics. We concede that in such cases, it may be reasonable to treat the nodal dynamics as pure integrators (systems with infinite time constants). However, we argue that structural controllability may not be appropriate for addressing these nuanced modeling issues. An essential feature of structural controllability is that no importance is assigned to *specific values* for the non-zero terms in the dynamics. Values are treated as either zero or not zero; there is no in-between. Thus, the choice of whether to zero out the self-loop terms *a priori* is a subtle modeling issue that should take into account the *emergent timescales of the entire network*. Therefore, we contend that model reduction [23]–which is essential for controller design–should be treated at the level of the entire network dynamics rather that at the level of individual nodes: indeed the timescales relevant for control are an emergent property of the *system* dynamics, and not strictly a feature of one node or another. With this in mind, we find that the tool of structural controllability–which is premised on a notion of generic parameters–is best suited to generic modeling assumptions. In this case this means assuming 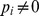, 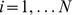.

**Figure 1:**
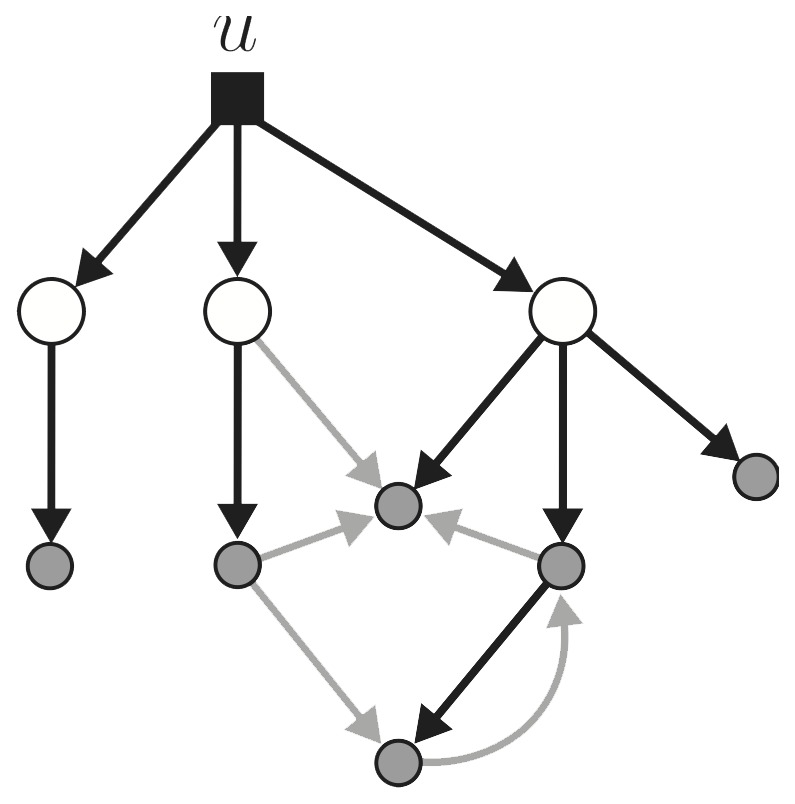
Given a network, the PDS (large white circles) is the smallest set of nodes such that all other nodes (smaller grey circles) are downstream of them. Any network, with arbitrary (and possibly different) order finite-dimensional linear dynamics at each node is structurally controllable from a single driver node (black square) tied to the PDS as shown. See Proposition 2. The edges in the *structural control network* are part of a minimum spanning tree (black edges, although this choice of edges, and indeed the PDS, is not necessarily unique).

Above, we argue that structural controllability of complex networks depends on the dynamics at each node, and that only a single time varying input is required. Two questions remain: (1) How sensitive is structural controllability to the dimension of the state space for each node? (2) Where should we inject the 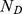 independent time inputs into the network, i.e. what is the minimum number of nodes of the network to which the input must be connected? Proposition 1 explicitly depends on treating first order nodal dynamics as “self loops” in the network. Below we offer a more general treatment for arbitrary (linear) nodal dynamics that addresses both questions above. See Figure 1.

Given a directed graph, a PDS is, by definition, the smallest set of nodes such that all other nodes are downstream of at least one node in the PDS. Obviously, controllability requires connecting the input(s) at least to this set; below we show that structural controllability is generically achieved by connecting a single input to the PDS. Before doing this, we need one definition:

**Definition 1** Suppose that there are 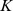 nodes in the PDS. Attach a single control input, 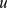, to this set via a control node. Augment the graph with this control node and add the 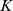 edges that connect it to the PDS. Then, all nodes are downstream of the input 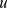 (i.e. the control node is now the PDS of the augmented graph). Define the **structural control network** as an acyclic directed graph given by a directed spanning tree that starts at 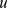 and visits all nodes.

We now state the main result.

**Proposition 2** Consider the nodal dynamics in (2), with 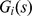 an arbitrary, proper, rational transfer function [15] of the form.
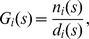
where, 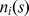 and 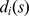 are assumed to be generic polynomials (all coefficients up to the order of the polynomial are assumed to be nonzero) of finite but arbitrary order in 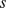. Then, the network is structurally controllable with one (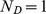) independent input, connected to the PDS.

*Proof.* Using the structural controllability argument, we are free to modify any nonzero parameters; if the system is controllable for one set of parameters, it will be generically controllable.

So, zero out all edges that are not in the structural control network and set all those in the structural control network to 1; if this process results in a controllable system, as we now show it does, then the system will be controllable generically.

All nodes in this structural control network are still downstream of 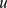, but now there are no cycles. Since the structural control network is a minimum spanning tree, there is exactly one path between 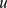 and any specific node, 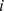. Let 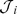 denote the set of nodes along the path from 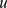 to node 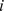 in the structural control network. Then transfer function from 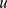 to any given node is simply the product of the transfer functions along the path from 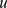 to the node:
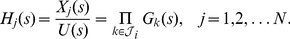
(5)


Since we may freely adjust the polynomial coefficients in the denominator terms, we do so to ensure there are no repeated poles in the entire network and similarly adjust the numerator coefficients to ensure no pole-zero cancellations along any path in the structural control network. Since there are no pole-zero cancellations, and all poles in the network are unique, a minimal realization of the 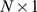 transfer function 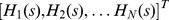 must contain exactly one eigenvalue for each pole of the network. It is obvious that the minimal realization requires no more eigenvalues than that. The number of eigenvalues in the minimal realization is equivalent to the number of eigenvalues that are both controllable and observable. Thus all states are controllable for this parameter set and, by the structural controllability theorem [11], the network is structurally controllable.

For first-order nodal dynamics, our main result is not substantively different from those presented for discrete time and finite state systems in [24], [25]. They show that networks with nontrivial nodal dynamics are structurally observable with a single output node and structurally controllable with a single input node. Our modest generalization to arbitrary-order nodal dynamics is at best incremental over their work. Indeed, the main contribution of our paper lies not so much in any technical advance as it does in providing a timely clarification of [10].

### Simple Example: A Food Web

To illustrate the ideas of this paper, consider a simple food web comprising one predator and one prey species. Let 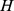 denote the number of herbivores (prey) and 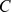 denote the carnivores (predators). We begin by noting that historically, the classic models of predator–prey dynamics [26] take the form.
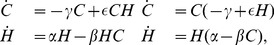
(6)where 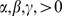. Linearizing these dynamics about the nontrivial equilibrium 
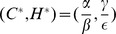
 this model has the following local dynamics:



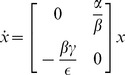
where 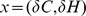 is the vector of small displacements relative to the equilibrium 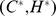. Note that the linearized food web is fully connected (whereas [10] include a nonzero edge for “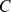 eats 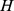
” but not for “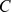 is eaten by 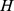
” in their treatment of a trophic networks). Also, note that this linearized system has infinite time constants at the nodes, i.e.,zero values along the diagonal. Thus these early models do not include the finite time constants that we argue are so important to system dynamics. Later work remedied this omission; the early models such as Eq. (6) did not include terms that researchers subsequently found to be essential for modeling real biological systems, such as saturation effects arising from resource limitations [14]. Including these additional terms leads to a 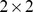 system matrix that is fully populated with (generically) nonzero terms on and off the diagonal. This implies that the resulting linearization features finite time constants at each of the nodes, and the network is fully connected. That is, where structural controllability is concerned, taking into account the full dynamics of a food web leads inescapably to the conclusion this system should be controllable with a single input.

## Discussion

Recently, it was reported that sparse inhomogenous networks require distinct controllers for a large fraction of the nodes to attain structural controllability [10]. We argue that these results are a consequence of assuming a special structure for the dynamics at each node: each node is treated as a pure integrator. In the application of the model set forth in [10] to the real networks considered therein, each node is assumed to have an infinite time constant. In this paper, we show that (1) for generic, arbitrary-order nodal dynamics, structural controllability can be achieved with a single time-varying input, and (2) that input should be attached to a PDS.

The property of a system being controllable has two significant interpretations in control theory. First, if a system is controllable then it is possible to find an input to transfer any initial state to any final state in finite time. Second, if a system is controllable then it is possible to apply a control signal consisting of a linear combination of the states that changes the dynamics arbitrarily. In particular, it is possible to stabilize an unstable system, a necessary design goal in engineering problems. Such a control signal is termed state feedback.

It is important to note what the first definition of controllability leaves out. For example, unless the final state is an equilibrium, the state will not remain there, but will move away. In many engineering applications, it is important to find an input that will both stabilize a system and hold a specified linear combination (or set of linear combinations) of states at desired constant values. This is referred to as the problem of setpoint tracking, and requires that the system be controllable (so that a stabilizing control input may be found) and that there are at least as many independent control inputs as there are linear combinations of states to be held at desired setpoints [27]. Hence we see that although one input may suffice to achieve controllability of an arbitrary number of state variables, in fact the number of inputs limits the number of setpoints that may be specified.

The property of controllability is generically present in a system, and thus in practice it is more important to know not whether a system is controllable, but whether it is almost uncontrollable. In the latter case, the control input used to drive the state to its desired value, or to achieve the desired dynamics, may be excessively large. Hence there is a need for tests–such as those based on the control Gramian [15]–to determine what states are almost uncontrollable. In practice these are then treated as though they were indeed uncontrollable to avoid the excessively large inputs required to control them.

A more subtle problem arises with the second use of the controllability property. In practice, it is rarely possible to measure all the states of the system required for the control signal used to alter the dynamics of the system. Instead, the control signal is based on estimates of the states obtained by processing those states (or linear combinations of states) that are measurable. A system is said to be observable if it is possible to estimate the states using only the available outputs [15]. As is the case with controllability, the property of observability is generically present, and it is necessary to determine whether states are almost unobservable.

States that are either uncontrollable or unobservable do not influence the input–output relation of a system, and cannot themselves be influenced by a control input signal based on output measurements. Such systems are characterized by a pole (an eigenvalue of the matrix 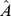) that does not appear in the transfer function due to being canceled by a zero of the transfer function having the same value. If the system is almost uncontrollable or almost unobservable, then the transfer function will have a zero very near to a pole. In this case, it is possible to design a control signal based on state estimates. However, it may be shown using the theory of fundamental design limitations [28], [29] that the resulting feedback control system will necessarily have a very small stability margin, and be sensitive to disturbances and parameter variations. Often, the solution to this problem requires the introduction of additional control inputs or additional measurements.

In conclusion, the property of controllability, although important, is by no means sufficient to assure a well behaved control problem. One might expect this to be true since the property is generically present, as is the property of observability. The more relevant questions are thus whether the system is almost uncontrollable, almost unobservable, or possesses almost pole–zero cancellations.
